# Hyperparasitoids Use Herbivore-Induced Plant Volatiles to Locate Their Parasitoid Host

**DOI:** 10.1371/journal.pbio.1001435

**Published:** 2012-11-27

**Authors:** Erik H. Poelman, Maaike Bruinsma, Feng Zhu, Berhane T. Weldegergis, Aline E. Boursault, Yde Jongema, Joop J. A. van Loon, Louise E. M. Vet, Jeffrey A. Harvey, Marcel Dicke

**Affiliations:** 1 Laboratory of Entomology, Wageningen University, Wageningen, the Netherlands; 2 Plant Ecology and Phytochemistry, Institute of Biology Leiden, Leiden University, Leiden, the Netherlands; 3 Department of Terrestrial Ecology, Netherlands Institute of Ecology (NIOO-KNAW), Wageningen, the Netherlands; Cornell University, United States of America

## Abstract

Plants respond to herbivory with the emission of induced plant volatiles. These volatiles may attract parasitic wasps (parasitoids) that attack the herbivores. Although in this sense the emission of volatiles has been hypothesized to be beneficial to the plant, it is still debated whether this is also the case under natural conditions because other organisms such as herbivores also respond to the emitted volatiles. One important group of organisms, the enemies of parasitoids, hyperparasitoids, has not been included in this debate because little is known about their foraging behaviour. Here, we address whether hyperparasitoids use herbivore-induced plant volatiles to locate their host. We show that hyperparasitoids find their victims through herbivore-induced plant volatiles emitted in response to attack by caterpillars that in turn had been parasitized by primary parasitoids. Moreover, only one of two species of parasitoids affected herbivore-induced plant volatiles resulting in the attraction of more hyperparasitoids than volatiles from plants damaged by healthy caterpillars. This resulted in higher levels of hyperparasitism of the parasitoid that indirectly gave away its presence through its effect on plant odours induced by its caterpillar host. Here, we provide evidence for a role of compounds in the oral secretion of parasitized caterpillars that induce these changes in plant volatile emission. Our results demonstrate that the effects of herbivore-induced plant volatiles should be placed in a community-wide perspective that includes species in the fourth trophic level to improve our understanding of the ecological functions of volatile release by plants. Furthermore, these findings suggest that the impact of species in the fourth trophic level should also be considered when developing Integrated Pest Management strategies aimed at optimizing the control of insect pests using parasitoids.

## Abstract 2

Parasitic wasps that develop inside herbivorous hosts alter the volatiles produced by plants in response to the damage, thus giving away the presence of the parasitoid larvae to their hyperparasitoid enemies.

## Author Summary

In nature, plants often release volatiles in response to damage by herbivores (e.g., by caterpillars), and these can indirectly help defend the plants. Indeed, it is well documented that volatiles can recruit the natural enemies of herbivores, such as predators and parasitoid wasps, whose offspring feed on and develop within their caterpillar hosts. However, such induced plant odours can also be detected by other organisms. One important group of organisms, hyperparasitoids, the enemies of the parasitoids that indirectly benefit the plants, have not been included in this trophic web because so little is known about their foraging behaviour. Here, using a combination of laboratory and field experiments, we demonstrate that hyperparasitoid wasps also take advantage of the odours that plants produce in response to the feeding by caterpillars. The larvae of parasitic wasps developing inside the caterpillar alter the composition of the oral secretions of their herbivorous host and thereby affect the cocktail of volatiles the plant produces. The hyperparasitoids on the lookout for their parasitoid prey can preferentially detect infected caterpillars, although not all parasitoid wasps gave away their presence through this host–plant interaction. We conclude that herbivore-induced plant volatiles can affect the interaction among parasitoids and their enemies and thereby may reduce the indirect defence accrued for the plant.

## Introduction

Plant volatiles play a profoundly important role in the structure and function of ecological communities [1]–[6]. Volatiles make a plant and its condition apparent to community members at different trophic levels [6],[7] and may, thereby, mediate interactions between organisms at higher trophic levels [8],[9]. Nowhere has this been better investigated than for interactions between insect herbivores and their natural enemies, such as primary parasitic wasps (or “parasitoids”) at the third trophic level. Many parasitoids have evolved finely tuned responses to volatiles emitted by plants that are attacked by their otherwise inconspicuous herbivorous hosts. By responding to volatiles and parasitizing the herbivores, parasitoids may reduce the amount of herbivory that plants are exposed to [1]–[3] and are, therefore, hypothesized to benefit plant fitness [10]–[12]. However, besides attracting beneficial parasitoids, the volatiles affect interactions between plants and other community members that may affect the fitness benefit of volatile release. Food webs generally include four or more trophic levels [13],[14]. Thus far, little is known about foraging behaviour of the enemies of parasitoids (i.e., hyperparasitoids) that are an important group of fourth-trophic-level organisms, because hyperparasitoids have not been included in the debate on the fitness benefit of volatile release by plants [15].

Hyperparasitoids are parasitic wasps that attack the larvae and pupae of primary parasitoids, and they comprise a major component of the fourth trophic level in insect communities [15]. Thus far, little is known about the cues that hyperparasitoids use to find their primary parasitoid hosts [15],[16]. For secondary hyperparasitoids (i.e., hyperparasitoids that attack the fully cocooned pupae of primary parasitoids), their hosts are likely to be inconspicuous because the pupae do not feed and, therefore, do not indirectly reveal their presence through induced volatiles of the food plant. Furthermore, the time window for successful hyperparasitism of pupae is often narrow and restricted to the first few days after the pupae are formed [17]. However, plants have been shown to respond differently to feeding damage inflicted by parasitized or unparasitized herbivores [18]–[20]. As a result, plant-derived volatile cues may provide hyperparasitoids with reliable information on the presence of their host [15].

Many hyperparasitoid species parasitize the pupae of a range of primary parasitoid species, including both solitary species, which lay a single egg within an herbivore, and gregarious species, which lay multiple eggs within a single herbivore [21]. Single or multiple parasitoid larvae developing in a caterpillar differentially affect the physiology and feeding behaviour of the herbivore, thereby inducing different plant volatile blends [19],[20]. Consequently, hyperparasitoids may be better able to locate one parasitoid host than the other, and thus variation in plant volatiles induced by parasitized herbivores may cause variation in the level of hyperparasitoid attack on different species of primary parasitoids. Here, we test whether plants can mediate interactions between third- and fourth-trophic-level organisms by providing cues on the presence of hosts for hyperparasitoids and whether hyperparasitoids respond similarly to herbivores that contain different parasitoids.

**Figure 1:**
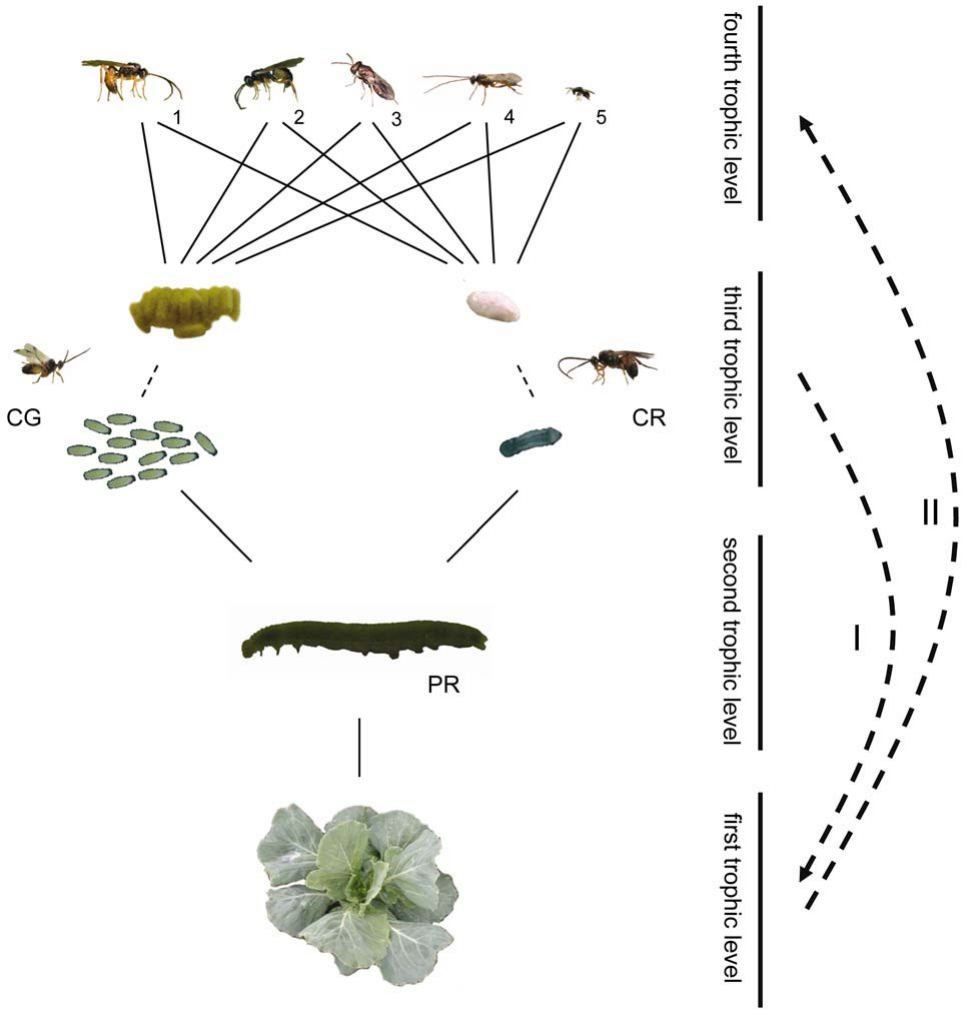
Experimental study system of the four-trophic-level community on *Brassica oleracea* plants. The gregarious primary parasitoid *Cotesia glomerata* (CG) and the solitary *C. rubecula* (CR) attack caterpillars of *Pieris* (PR) butterflies, which are in turn attacked by several hyperparasitoids: *Acrolyta nens* (1), *Lysibia nana* (2), *Pteromalus semotus* (3), *Mesochorus gemellus* (4), and *Baryscapus galactopus* (5). Hyperparasitoids at the fourth trophic level find their primary parasitoid host at the third trophic level via information derived from the plant at the first trophic level. Larvae of primary parasitoids that develop in their herbivorous host at the second trophic level inflict changes in their herbivore host, and the combination of herbivore and parasitoid (parasitized herbivores) inflict changes in plant volatile emission (I). These changes in plant volatile emission are used by hyperparasitoids as a cue of host presence (II). Photograph credit: Tibor Bukovinszky.

To study these questions we used the hyperparasitoid *Lysibia nana* (Ichneumonidae) that attacks pupae of primary parasitoids in the genus *Cotesia* (Hymenoptera: Braconidae). The solitary parasitoid *C. rubecula* (CR) and the gregarious *C. glomerata* (CG) are primary parasitoids that both attack caterpillars of the Small Cabbage White butterfly, *Pieris rapae* (PR), that feed on brassicaceous plants (Figure 1) [22]. When fully developed, the parasitoid larvae leave their host to spin a silk cocoon in which they pupate. Individual *C. glomerata* cocoons are approximately 40% smaller (in terms of mass) than individual *C. rubecula* cocoons. In terms of the *per capita* fitness potential of hyperparasitoid offspring, *L. nana* may benefit when developing in pupae of the larger *C. rubecula* [23]. However, in terms of cumulative maternal fitness, *L. nana* hyperparasitoids benefit more when finding a caterpillar parasitized by the gregarious *C. glomerata*. In the field, caterpillars parasitized by *C. glomerata* produced an average of 39 *C. glomerata* cocoons. Upon finding clusters of their primary parasitoid host, hyperparasitoids generally parasitize most or even all pupae in the brood, whereas in the case of finding a solitary primary parasitoid they have to disperse after each parasitization. The egg load of *L. nana* females closely approximates the average brood size (e.g., 20–40) of *C. glomerata* [17], suggesting that it co-evolved with gregarious host species such as *C. glomerata*. We hypothesized that *L. nana* uses volatile plant cues induced by *C. glomerata*–parasitized caterpillars to locate aggregated pupae and that they prefer those volatiles over volatile plant cues induced by the feeding of solitarily parasitized caterpillars.

## Results

**Figure 2:**
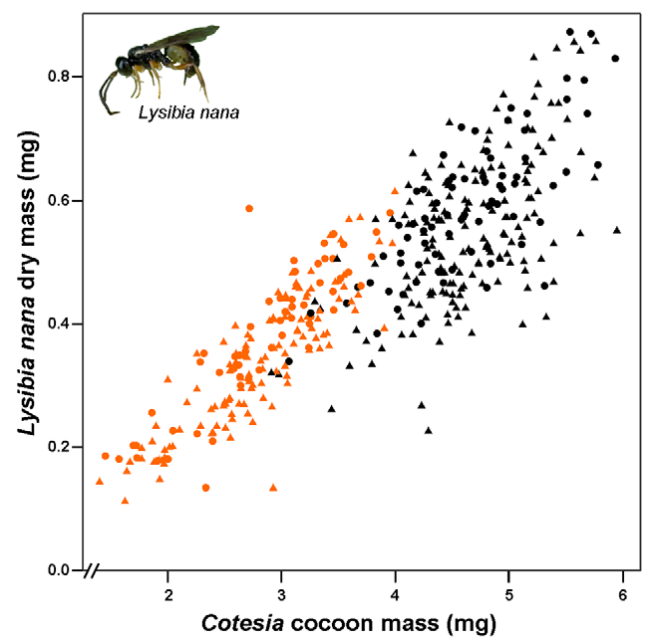
Performance of *Lysibia nana* on pupae of two parasitoid species. *Lysibia nana* dry mass plotted against the mass of the *Cotesia* cocoon before *L. nana* had parasitized the cocoon. Orange symbols represent wasps emerging from *C. glomerata* cocoons, and black symbols those emerging from *C. rubecula* cocoons. Females are represented by dots, and males by triangles. Photograph credit: Tibor Bukovinszky.

In line with differences in the size of their hosts, we found that *L. nana* wasps were about 35% smaller when emerging from *C. glomerata* than *C. rubecula* cocoons (Table 1; Figure 2).

**Table 1:** Fresh weight and clutch size of cocoons of two *Cotesia* species collected from a laboratory rearing or from the field, and the corresponding fitness-related traits in the hyperparasitoid *Lysibia nana*.

Wasp Parameter	*Cotesia glomerata*	*Cotesia rubecula*
Cocoon mass (laboratory) (mg)	2.84±0.59 (*n* = 179)	4.67±0.55 (*n* = 238)
Cocoon mass (field) (mg)	2.89±0.78 (*n* = 1128)	4.88±0.97 (*n* = 1553)
Clutch size (field) (nr. cocoons per host)	39.41±21.46 (*n* = 1,128)	

	Female (*n* = 58)	Male (*n* = 121)	Female (*n* = 75)	Male (*n* = 163)
*Lysibia nana* dry mass (mg)	0.38±0.12	0.34±0.11	0.59±0.11	0.55±0.12
Development time (hours)	341.29±25.48	329.65±24.57	344.00±21.15	335.99±19.82

Values indicate mean ± SD.

**Figure 3:**
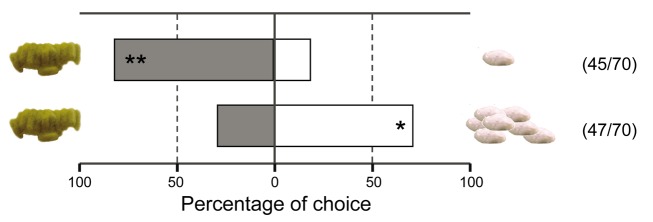
*Lysibia nana* responses in choice tests with primary parasitoid cocoons. *Lysibia nana* preference (top bar) for gregarious broods of *Cotesia glomerata* (grey) or solitary cocoons of *C. rubecula* (white) in a Petri dish bioassay. *Lysibia nana* preference (lower bar) for gregarious broods of *Cotesia glomerata* (grey) or the same number of cocoons of *C. rubecula* (white). Numbers between brackets indicate the fraction of wasps that responded to cocoons within 10 min from the start of the experiment. * *p*<0.05, ** *p*<0.001. Photograph credit: Tibor Bukovinszky.

When testing the response to volatiles directly derived from cocoons in a Y-tube olfactometer, *L. nana* wasps were not attracted to odours associated with the pupae, such as those derived from silk with which the wasps have spun a cocoon to house the pupa. Only 5% of the wasps tested walked up the olfactometer arm within 10 min. Therefore, we studied *L. nana* responses to cocoons when offered at a closer range, similar to conditions that hyperparasitoids may experience after landing on a plant. *L. nana* females were offered a choice between a brood of the gregarious parasitoid *C. glomerata* (CG) and a cocoon of the solitary wasp *C. rubecula* (CR) in a glass Petri dish (18.5 cm diameter, 4.6 cm height). When the hyperparasitoid wasps were released, we did not observe any directional movement towards the cocoons and the hyperparasitoid wasps often passed cocoons within a centimeter distance. Despite their activity and limited search area, within 10 min, 45 of the 70 tested wasps encountered one of the cocoons and were arrested. A cocoon clutch of *C. glomerata* was more commonly found than a solitary cocoon of *C. rubecula* (binomial test, *n* = 45, *p*<0.001) (Figure 3). When we offered cocoons of *C. rubecula* in a group, to match the cocoon mass of a brood of *C. glomerata* cocoons, we found that *L. nana* females more often first encountered the group of *C. rubecula* cocoons than the clutch of *C. glomerata* cocoons (*n* = 47, *p* = 0.003) (Figure 3). This may be due to the larger surface covered by the group of *C. rubecula* cocoons than the surface covered by the brood of *C. glomerata* cocoons.

### Response of Hyperparasitoids to Herbivore-Induced Plant Volatiles

**Figure 4:**
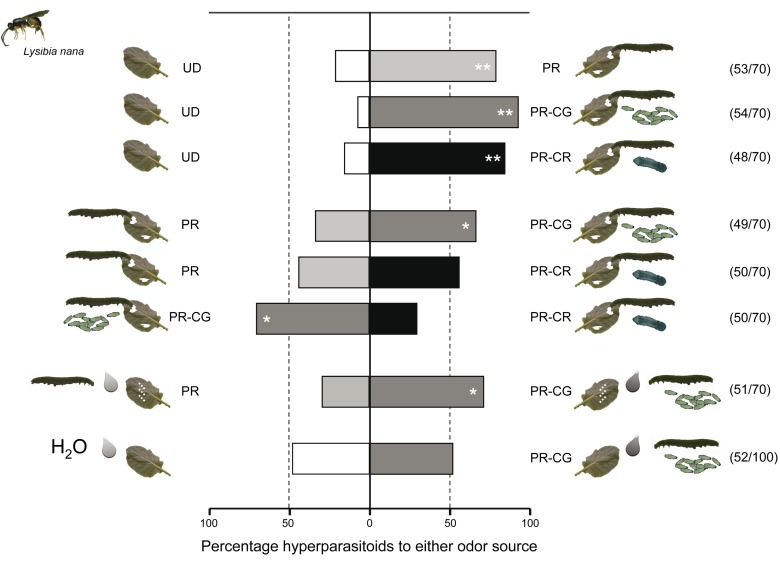
Preference of hyperparasitoids for herbivore-induced plant volatiles. Preference of the hyperparasitoid *Lysibia nana* for herbivore-induced plant volatiles was tested by using a full factorial design of two-choice olfactometer tests including pair-wise comparisons of the treatments: undamaged plants (white bars), *Pieris rapae* damaged plants (light grey), plants damaged by *Pieris rapae* caterpillars parasitized by *Cotesia glomerata* (dark grey bars), or plants damaged by *Pieris rapae* caterpillars parasitized by *C. rubecula* (black bars). The two lowest pairs of bars show the preference of *L. nana* for plants treated with caterpillar regurgitant. The first pair shows hyperparasitoid preference when plants are artificially damaged and regurgitant of unparasitized (light grey) or parasitized (dark grey) caterpillars was applied. The second and lowest pair shows that hyperparasitoids do not respond to the application of regurgitant without artificially damaging the plant. Numbers between brackets indicate the number of wasps that made a choice within 10 min from the start of the experiment versus the total number of wasps tested. * *p*<0.05, ** *p*<0.001. Photograph credit: Tibor Bukovinszky.

Hyperparasitoids did respond to herbivore-induced plant volatiles. *L. nana* females preferred volatiles from plants damaged by either caterpillars parasitized by primary parasitoids (both *C. glomerata* [PR-CG] and *C. rubecula* [PR-CR]) or unparasitized caterpillars (PR) over volatiles from undamaged plants in a Y-tube olfactometer (Figure 4; binomial tests, *p*<0.001). The hyperparasitoids did not discriminate between volatile blends from plants damaged by unparasitized caterpillars and plants damaged by caterpillars parasitized by the solitary parasitoid *C. rubecula* (Figure 4; binomial test, *p* = 0.480). However, plant volatiles induced by *C. glomerata*–parasitized caterpillars were more attractive to *L. nana* than volatiles from plants damaged by unparasitized caterpillars or those parasitized by the solitary parasitoid *C. rubecula* (binomial tests, *p* = 0.021 and *p* = 0.007, respectively) (Figure 4). Unparasitized and *C. glomerata*–parasitized caterpillars inflicted more damage to plants than caterpillars parasitized by a solitary *C. rubecula* parasitoid—that is, respectively, 2,485±1,183 (mean ± SD), 1,855±810, and 705±313 mm^2^ of leaf tissue consumed per caterpillar in 24 h (Kruskal-Wallis Test, *p*<0.001). Because parasitoid species differentially affect caterpillar feeding rate by regulating the growth of their host [19] and the rate of feeding damage per se may mediate the attraction of *L. nana*, we controlled for the amount of damage in a subsequent test. We damaged plants with a pattern wheel and applied 25 µl of oral secretion from either unparasitized or parasitized caterpillars to the damaged sites. Parasitoid species are known to alter the composition of the oral secretions of their host and thereby strongly affect the response of the plant to a parasitized caterpillar [20]. Compounds in the oral secretions of *Pieris* caterpillars play a key role in inducing volatile release by their food plant [24]. *L. nana* preferred the volatiles from plants that were treated with oral secretions obtained from *C. glomerata–*parasitized caterpillars over volatiles from plants treated with oral secretions from unparasitized caterpillars (Figure 4). Oral secretions of parasitized caterpillars alone (i.e., without application to wounded sites) did not attract the hyperparasitoids (Figure 4).

### Volatiles

**Figure 5:**
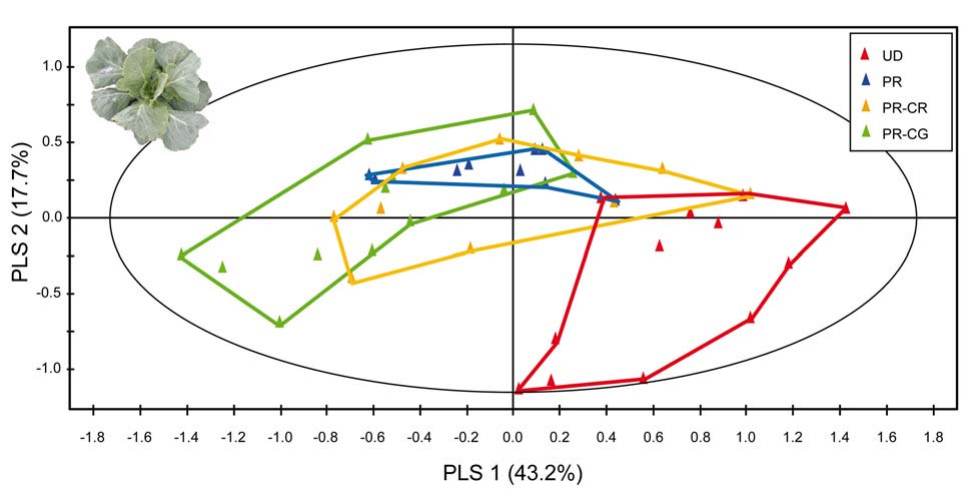
PLS-DA plot based on comparisons among volatile blends of *Brassica oleracea* plants under herbivory by parasitized or unparasitized caterpillars. Plants were either undamaged (red, UD), damaged with two unparasitized *Pieris rapae* caterpillars (blue, PR), or *P. rapae* caterpillars parasitized by *Cotesia rubecula* (orange, PR-CR) or *C. glomerata* (green, PR-CG). Photograph credit: Tibor Bukovinszky.

Analysis of the volatile blends of plants induced by *C. glomerata*–parasitized, *C. rubecula*–parasitized, or unparasitized caterpillars revealed that these three herbivore treatments induce volatile blends that differ from undamaged control plants. A total of 33 compounds that were present in all samples of at least one of the four plant treatments were tentatively identified and included in further analysis (Table 2). In the PLS-DA, undamaged control plants grouped separately from the three treatments with caterpillar feeding (Figure 5). Amongst the caterpillar-damage treatments, plants damaged by feeding of unparasitized caterpillars and caterpillars parasitized by *C. rubecula* overlapped largely in their volatile headspace as shown by PLS-DA. Plants damaged by *C. glomerata–*parasitized caterpillars were only 40% similar in their volatile headspace to plant headspaces induced by the two other caterpillar treatments and were most distinctly different from undamaged control plants. Nine compounds most strongly contributed to the differences among treatments are indicated by VIP scores higher than 1. These compounds included terpenoids, a ketone, a nitrile, and two unknown compounds (Table 2). The concentrations of two compounds differed significantly among the caterpillar treatments. Plants damaged by *C. glomerata–*parasitized caterpillars produced higher concentrations of (*E*)-4,8-dimethylnona-1,3,7-triene [(*E*)-DMNT], a known attractant for parasitoids [25], and of an unknown compound compared to plants damaged by *C. rubecula–*parasitized or unparasitized caterpillars. The similarity of the volatile blends from plants damaged by unparasitized and *C. rubecula*–parasitized *P. rapae* matches the observation that hyperparasitoids did not discriminate the two treatments in choice assays. The hyperparasitoids did prefer plants damaged by *C. glomerata*–parasitized caterpillars over other damage treatments, which is supported by the difference in the composition of the volatile blends emitted by the plants submitted to these treatments.

**Table 2:** Volatile compounds detected in the headspace of *Brassica oleracea*, uninfested (control), or infested with two caterpillars of *Pieris rapae* that were either unparasitized or parasitized with *Cotesia rubecula* or *C. glomerata*.

Compound	Retention Time	Class	Control	*Pieris rapae*	*Pieris rapae* (*C. rubecula*)	*Pieris rapae* (*C. glomerata*)	VIP Score
(*Z*)-3-hexen-1-ol	9.98	Alcohol	78.92 (10.32)	79.09 (6.03)	76.26 (8.84)	95.10 (7.80)	0.61
phenylethyl-alcohol	18.66	Alcohol	1.12 (0.09)^a^	3.17 (0.54)^b^	2.76 (0.40)^b^	4.50 (0.74)^b^	**2.07**
1-penten-3-one	5.07	Ketone	2.35 (0.37)	1.91 (0.12)	1.83 (0.21)	2.12 (0.22)	0.73
3-methyl-2-pentanone	6.83	Ketone	1.37 (0.13)^a^	2.41 (0.22)^b^	2.43 (0.24)^b^	3.75 (0.50)^b^	**1.49**
2-cyclopenten-1-one	9.31	Ketone	4.05 (0.72)	4.56 (0.67)	3.50 (0.47)	4.71 (0.59)	0.60
6-methyl-2-heptanone	13.28	Ketone	7.62 (0.76)	6.45 (0.42)	6.59 (0.57)	7.90 (0.76)	0.73
α-thujene	12.53	Terpenoid	84.43 (5.63)	101.54 (5.53)	109.79 (7.92)	115.33 (4.58)	0.52
α-pinene	12.83	Terpenoid	52.18 (3.03)	54.88 (2.27)	57.19 (3.43)	61.74 (2.45)	0.33
sabinene	14.15	Terpenoid	290.94 (16.69)	353.06 (16.20)	374.16 (23.87)	389.78 (12.29)	0.49
β-pinene	14.34	Terpenoid	218.42 (12.34)	241.70 (11.40)	252.54 (15.50)	271.27 (9.71)	0.37
β-myrcene	14.53	Terpenoid	396.71 (18.00)	429.14 (16.95)	455.57 (28.96)	483.89 (19.80)	0.35
α-phellandrene	15.17	Terpenoid	29.75 (3.44)	29.19 (2.67)	33.15 (3.69)	36.49 (3.72)	0.62
α-terpinene	15.57	Terpenoid	64.66 (7.04)	62.52 (5.76)	69.10 (7.42)	77.56 (8.36)	0.61
limonene	16.00	Terpenoid	285.70 (15.86)	328.26 (15.70)	355.34 (28.00)	382.82 (17.27)	0.47
β-phellandrene	16.05	Terpenoid	29.15 (3.72)	25.38 (2.17)	25.41 (2.58)	32.16 (3.91)	0.78
1,8-cineole	16.14	Terpenoid	176.30 (11.34)	216.82 (11.71)	239.58 (19.20)	251.99 (11.24)	0.57
(*E*)- β-ocimene	16.41	Terpenoid	11.14 (0.98)	11.82 (0.83)	13.97 (1.59)	15.78 (1.20)	0.70
γ-terpinene	16.90	Terpenoid	75.36 (7.46)	74.09 (6.39)	83.75 (9.19)	94.22 (9.12)	0.60
dihydromyrcenol	17.13	Terpenoid	17.61 (4.18)^a,b^	11.67 (2.20)^a^	21.93 (4.24)^a,b^	27.77 (5.39)^a,b^	**1.27**
(*E*)-4-thujanol	17.24	Terpenoid	1.00 (0.09)^a^	1.71 (0.15)^b^	1.90 (0.17)^b^	1.87 (0.15)^b^	**1.32**
p-mentha-2,4(8)-diene	17.90	Terpenoid	48.70 (4.28)	50.91 (3.84)	56.43 (5.75)	62.04 (5.09)	0.53
linalool	18.08	Terpenoid	0.79 (0.14)	0.65 (0.04)	0.78 (0.09)	1.10 (0.20)	0.94
(*E*)-DMNT*	18.56	Terpenoid	5.50 (0.64)^a^	7.49 (1.08)^b^	6.70 (1.21)^a,b^	15.18 (3.33)^c^	**1.37**
terpinen-4-ol	20.78	Terpenoid	3.64 (0.32)	4.04 (0.21)	4.88 (0.47)	5.36 (0.42)	0.65
geranyl linalool	40.37	Terpenoid	1.74 (0.17)^a^	3.59 (0.37)^b^	3.41 (0.44)^b^	4.57 (0.44)^b^	**1.57**
(*Z*)-2-penten-1-ol acetate	11.81	Ester	25.22 (3.17)	26.10 (2.27)	27.14 (4.23)	36.53 (4.13)	0.81
(*Z*)-3-hexen-1-ol acetate	15.00	Ester	767.97 (44.11)	792.14 (31.63)	823.81 (64.61)	969.04 (54.77)	0.49
hexyl acetate	15.15	Ester	23.12 (1.95)	25.75 (2.09)	27.18 (3.51)	35.55 (3.24)	0.77
n-heptyl acetate	18.33	Ester	3.23 (0.23)	3.28 (0.19)	3.57 (0.43)	4.19 (0.42)	0.66
dimethyl disulfide	6.70	Sulfide	26.29 (6.41)	17.54 (2.26)	17.75 (1.93)	24.42 (4.48)	0.91
2,4-pentadienenitrile	7.46	Nitrile	0.56 (0.20)^a^	0.21 (0.02)^a^	0.29 (0.06)^a^	0.58 (0.16)^a^	**1.99**
Unknown	4.45	Unknown	0.99 (0.25)^a^	2.20 (0.57)^b,c^	1.37 (0.18)^b^	2.41 (0.29)^c^	**1.60**
Unknown	41.63	Unknown	13.67 (1.55)^a^	28.61 (3.40)^b^	26.95 (3.85)^b^	36.26 (4.01)^b^	**1.59**

Only those compounds have been included that were present in all replicates of at least one treatment. Amounts of individual compounds are given as average peak area (SE) per litre of trapped air per gram shoot biomass. Variable Importance in the Projection (VIP) scores for the PLS-DA are given. Bold face type scores are higher than 1 and are most influential for separation of the treatments. Differences among treatments for compounds with VIP score >1 based on Mann–Whitney U pair wise comparisons are indicated with superscript letters.

* (E)-DMNT = (E)-4,8-dimethylnona-1,3,7-triene.

### Hyperparasitoid Responses in the Field

**Figure 6:**
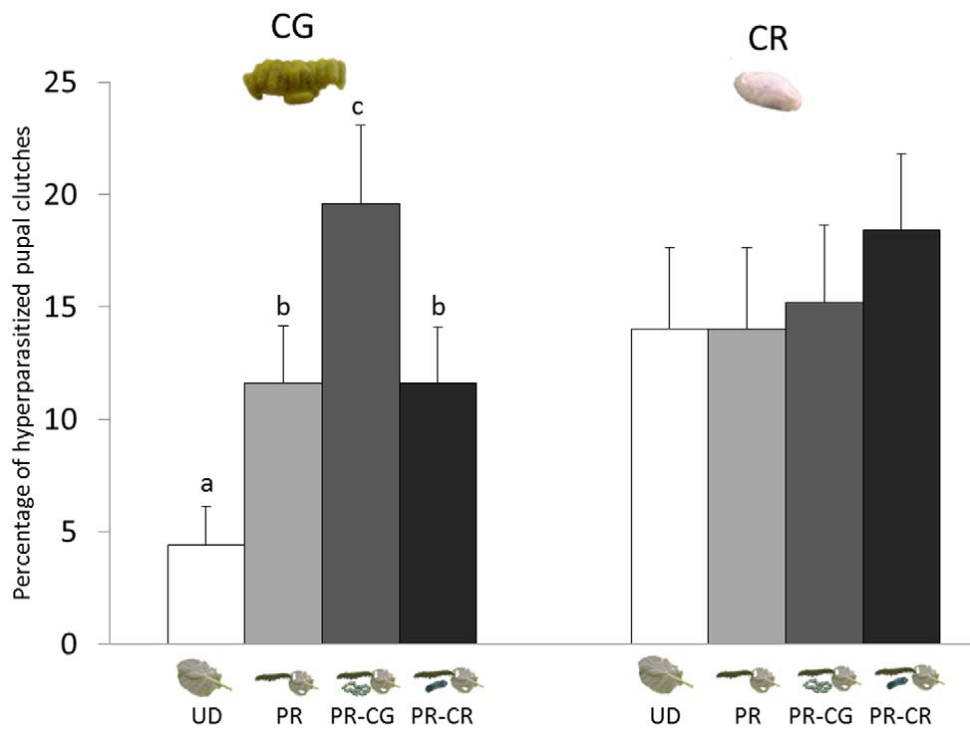
Herbivore-induced plant volatiles mediate hyperparasitism in the field. Percentage of *Cotesia glomerata* (CG, left) and *C. rubecula* (CR, right) cocoon clutches hyperparasitized on plants that had been induced with herbivory by unparasitized or parasitized caterpillars of *P. rapae*. *Pieris rapae* (PR), *P. rapae* parasitized by *C. glomerata* (PR-CG), *P. rapae* parasitized by *C. rubecula* (PR-CR), and undamaged (UD). Letters indicate significant differences between treatment groups (GLM, *p*<0.05). Photograph credit: Tibor Bukovinszky.

In the field, we confirmed that plant volatiles play an important role in the location of parasitoid pupae by hyperparasitoids. In an experimental field, *B. oleracea* plants were subjected to four induction treatments: no damage (UD), feeding by healthy *P. rapae* caterpillars (PR), or feeding by *P. rapae* caterpillars parasitized by either *C. rubecula* (PR-CR) or *C. glomerata* (PR-CG). After the caterpillars had fed on the plants for 10 d, which was approximately the total development period of the *Cotesia* larvae, the caterpillars were removed. On half of the plants per treatment, we then attached *C. glomerata* cocoons and on the other half *C. rubecula* cocoons. The cocoons were exposed to the natural population of hyperparasitoids and recollected to assess the number of cocoons that was hyperparasitized. *C. glomerata* pupae that were attached to plants damaged by *C. glomerata*–parasitized caterpillars were more frequently hyperparasitized than pupae attached to plants damaged by unparasitized or *C. rubecula*–parasitized caterpillars (Figure 6, Table 3). However, when *C. rubecula* cocoons were used to assess hyperparasitism rates, we found no induction treatment effect.

**Table 3:** The effect of plant induction treatment on the fraction of primary parasitoid cocoons per plant that contained any hyperparasitoid in the field.

Model Factor	Deviance	Degree of Freedom	*p* Value
Full model	797.45	399	
Factor			
Caterpillar induction (1)	16.00	3	**0.001**
Replicate (2)	258.83	4	**<0.001**
Type of cocoons (3)	5.53	1	**0.019**
Interaction			
1×2	33.48	12	**<0.001**
1×3	16.94	3	**<0.001**
2×3	10.71	4	**0.030**
1×2×3	11.17	12	0.514

Boldface type presents significant effects (α = 0.05) in a GLM model with a binomial distribution.

**Figure 7:**
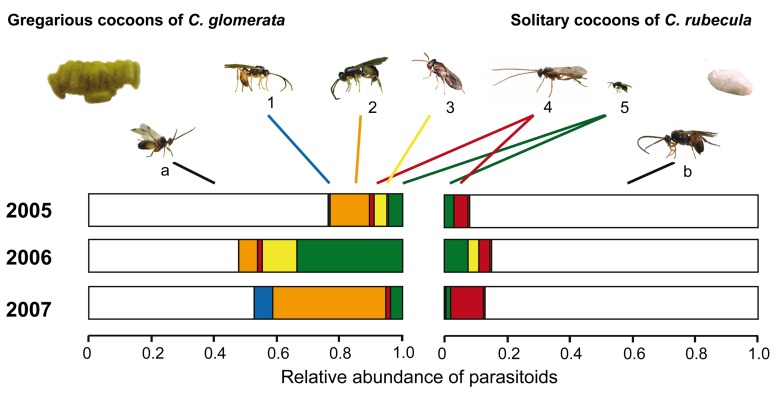
Relative abundance of primary parasitoids and hyperparasitoids from primary parasitoid cocoons in the field. Primary and hyperparasitoid wasps were reared from cocoons of the gregarious *Cotesia glomerata* (left) and solitary *C. rubecula* (right) that had been collected from *Brassica oleracea* during three field seasons. Colors indicate the different parasitoid species; the white segment of the bars depicts the primary parasitoids (a) *C. glomerata* and (b) *C. rubecula*; colored bar segments represent the most abundant hyperparasitoids: *Acrolyta nens* (1, blue bar), *Lysibia nana* (2, orange bar), *Pteromalus semotus* (3, yellow bar), *Mesochorus gemellus* (4, red bar), and *Baryscapus galactopus* (5, green bar). Photograph credit: Tibor Bukovinszky.

The preference of *L. nana* for volatiles derived from plants damaged by *C. glomerata*–parasitized caterpillars has profound consequences for the primary parasitoid *C. glomerata* in the field. During the growing season of cabbage plants in 3 consecutive years in the vicinity of Wageningen, the Netherlands, we collected 1,256 cocoon clusters of the gregarious primary parasitoid *C. glomerata* and 1,668 cocoons of the solitary primary parasitoid *C. rubecula* and assessed the rate of natural hyperparasitoid attack. Clusters of *C. glomerata* cocoons more often contained at least a single hyperparasitoid than did solitary cocoons of *C. rubecula* (Generalized Linear Model, deviance = 496.62, *p*<0.001; Table 4). From 17.4% of the *C. glomerata* clusters, more than one (and occasionally even four) hyperparasitoid species emerged. Hyperparasitoid communities associated with the gregarious primary parasitoid also consisted of more species than were found on the solitary parasitoid (Figure 7; Table 5). Within clusters of *C. glomerata* cocoons that were attacked by hyperparasitoids, 65%–81% of the pupae in the cluster yielded hyperparasitoid wasps. The combined attack rate of clusters and the fraction of pupae hyperparasitized in a cluster resulted in a total hyperparasitism rate of individual *C. glomerata* pupae of 20%–55% over the 3 y (Figure 7). By contrast, only 5%–15% of individual *C. rubecula* cocoons were hyperparasitized over the 3 y of our field experiments. Gregarious *C. glomerata* are, therefore, not only more easily found by hyperparasitoids, but once found, the hyperparasitoid wasps are able to parasitize large numbers of parasitoid pupae within broods, revealing that they exhibit an aggregative response to this clustered resource.

**Table 4:** The effect of *Cotesia* species and year on the fraction of hyperparasitism in field experiments carried out in 3 consecutive years.

Full Model	*Cotesia*	species		Year	*Cotesia ×*	Year
Deviance	Deviance	*p*	Deviance	*p*	Deviance	*p*
3,339.92	492.62	**<0.001**	178.38	**<0.001**	55.59	**<0.001**

Boldface type presents significant effects at *p*<0.001 in a GLM model with a binomial distribution.

**Table 5:** Hyperparasitoid species and the number of hyperparasitoid wasps emerging from *Cotesia glomerata* and *C. rubecula* cocoons collected during a 3-year survey.

Hyperparasitoid Species	Family	Subfamily	Parasitism Mode	*Cotesia glomerata* 2 (*n* = 1,256)	*Cotesia rubecula* (n = 1,668)
*Acrolyta nens*	Ichneumonidae	Cryptinae	Secondary	839	1
*Lysibia nana*	Ichneumonidae	Cryptinae	Secondary	9,923	8
*Gelis agilis*	Ichneumonidae	Cryptinae	Secondary	60	1
*Gelis acororum*	Ichneumonidae	Cryptinae	Secondary	3	—
*Bathythrix aerea*	Ichneumonidae	Cryptinae	Secondary	24	—
*Mesochorus gemellus*	Ichneumonidae	Mesochorinae	Primary	538	82
*Pteromalus semotus*	Braconidae	Hexothecinae	Secondary	1,457	28
*Pteromalus chrysos*	Braconidae	Hexothecinae	Secondary	38	—
*Baryscapus galactopus* 1	Eulophidae	Tetrastichinae	Primary	4,681	79

The numbers represent individual cocoons of the two parasitoids from which either a primary parasitoid or a hyperparasitoid emerged.

1 *Baryscapus galactopus* is a gregarious hyperparasitoid that develops with on average eight individuals in a single *C. rubecula* cocoon. The numbers for *B. galactopus* represent individual *Cotesia* cocoons that were hyperparasitized by *B. galactopus* (and thus on average produced eight hyperparasitoids). The number of cocoons of the gregarious *C. glomerata* that were hyperparasitized was calculated by dividing the total number of emerging *B. galactopus* by eight.

2 Number indicates the collected number of cocoon clutches, from which 25,170 *C. glomerata* wasps emerged.

## Discussion

Our results show that hyperparasitoids use plant volatiles to locate cocoons of their parasitoid host and that a network of interactions between the parasitoid, herbivore, and its food plant is involved in providing hyperparsitoids with cues of host presence. Interestingly, only one of the two parasitoids studied here altered the response of its herbivorous host with the food plant and thereby gave away its presence to hyperparasitoids. This gregarious parasitoid species, *C. glomerata*, was most frequently attacked by hyperparasitoids in the field, indicating that plant volatiles differentially expose parasitoid species to their enemies.

The two primary parasitoids that are suitable hosts for the hyperparasitoid *L. nana* largely differ in how they interact with their herbivorous host. *C. glomerata* larvae dynamically regulate the growth of their host in accordance with brood size and the concomitant amount of resources necessary to maximize adult parasitoid body size [26]–[28]. In contrast, *C. rubecula*–parasitized caterpillars are developmentally arrested in the third or fourth instar, depending on instar parasitized, and consume much less plant tissue than healthy caterpillars [19],[29]. However, the amount of feeding damage per se did not explain that *L. nana* discriminated *C. glomerata*–parasitized from unparasitized caterpillar-damaged plants. Controlling for the amount of damage and application of oral secretions of caterpillars revealed that changes in the oral secretions underlie the effect on differential plant responses to parasitized and healthy caterpillars. The oral secretions of parasitized caterpillars can be visually distinguished in terms of colour from oral secretions of healthy caterpillars, and several parasitoid species have different effects on the colour of oral secretions [20]. Clearly, the oral secretions differ in composition among caterpillars parasitized by different parasitoids, and the nature of those changes merits further investigation. Nevertheless, application of oral secretions of unparasitized or parasitized caterpillars to plant wounds has been found to result in differential expression of genes underlying the herbivore-induced plant volatile emission [20]. Here, we show that differences in induced responses of plants to caterpillars in which different parasitoids develop results in different blends of volatiles produced by the plant. However, these induced changes in volatiles were only present when *C. glomerata* had parasitized the herbivore and not when *C. rubecula* parasitized the same herbivore species. This may be caused or confounded by the lower amount of damage that *C. rubecula*–parasitized caterpillars make when compared to *C. glomerata*–parasitized caterpillars. Alternatively, it may suggest that *C. rubecula* has evolved to reduce its conspicuousness by not affecting the elicitors in oral secretions of its host that may reveal its presence to its hyperparasitoid enemies, whereas *C. glomerata* does reveal itself through such changes. In its introduced range in the United States, *C. rubecula* is frequently hyperparasitized [21]. This may suggest that in its invasive range *C. rubecula* has not (yet) adapted to be inconspicuous when developing in *P. rapae*, because of the shorter co-evolutionary relationship between *P. rapae* and *C. rubecula* in the invasive range. Because of the differences in attraction of hyperparasitoids to volatiles emitted by plants induced with caterpillars parasitized by different parasitoid species, we hypothesize that plant responses to herbivory result in differential selection pressure of hyperparasitoids on primary parasitoids. The observation of variation in the attraction of hyperparasitoids to caterpillars parasitized by different parasitoids that are suitable hosts to the hyperparasitoid also raises the question of whether hyperparasitoids may be able to use plant odours to assess whether a herbivore is parasitized by a nonsuitable parasitoid host. This area certainly merits further investigation.

### Fitness Consequences of Response to Delayed Rewards

Secondary hyperparasitoids that are attracted to volatiles emitted by plants that are damaged by parasitized caterpillars containing fully grown parasitoid larvae may suffer fitness costs by arriving too early on a plant (i.e., before the larvae of the their primary parasitoid hosts have emerged and constructed cocoons). However, pupae themselves do not interact with plants, and plants on which no active feeding takes place are likely to have reduced volatile emission and are, therefore, more difficult to detect [1]. In addition, hyperparasitoids are even further constrained in host searching as they can only successfully parasitize young pupae of primary parasitoids (i.e., within several days after they are formed) [30]. Therefore, hyperparasitoids are likely to have evolved to respond to highly detectable and reliable cues that predict the presence of available pupae in the near future and require hyperparasitoids to wait, rather than responding to cues that may result in arriving too late.

Similar waiting strategies have been observed for pupal parasitoids that are similarly constrained in terms of host suitability (i.e., they can only parasitize the pupae of herbivores shortly after these are formed). We have observed on numerous occasions that the pupal parasitoid *Pteromalus puparum* sits on or next to final instar *Pieris* caterpillars awaiting their pupation (E.H. Poelman and J.A. Harvey, unpublished observations). For both hyperparasitoids and pupal parasitoids, natural selection may favour such a waiting strategy even further, because healthy and parasitized caterpillars are well known to leave the host plant during the wandering phase and to climb onto a neighbouring plant and are thus not detectable through volatiles emitted by the plant on which they were previously feeding. The strategy of herbivores or parasitized herbivores to wander off the plant on which they have been feeding suggests that selection is being imposed on such behaviour, which is likely to be mediated by responses of their own natural enemies such as hyperparasitoids.

Despite the potential delay of arriving too early and having to wait on the plant to be able to parasitize the primary parasitoid pupae, the plant volatile cues derived from feeding by parasitized herbivores are the most detectable cues predicting host presence. *L. nana* females generally carry up to 40 mature eggs after several days that are ready for oviposition and thus can exploit an entire brood of *C. glomerata* within several hours [30]. Moreover, a female can mature an additional 20–30 eggs over the course of 24 h [30]. This suggests that *L. nana* has probably co-evolved with host species such as *C. glomerata* because of the strong synchrony between egg load dynamics in the hyperparasitoid and average cocoon cluster size in *C. glomerata* [31].

### Foraging Decisions on the Food Plant

Foraging decisions of the hyperparasitoids on the food plant clearly underlie the contrast between *C. glomerata* and *C. rubecula* hyperparasitism levels when cocoons of *C. glomerata* and *C. rubecula* were exposed to the natural hyperparasitoid community on plants damaged by parasitized or healthy caterpillars.

The field study in 2011 showed a preference of hyperparasitoids for plants damaged by gregariously parasitized caterpillars, and this was reflected in hyperparasitism rates on gregarious *C. glomerata* pupae (Figure 6), supporting our findings in the laboratory choice assays. However, the effects of the herbivory treatments in the field assay did not prevail on solitary pupae of *C. rubecula*. Moreover, we also found a higher hyperparasitism rate of *L. nana* in the solitary pupae in the field season of 2011 compared to the field seasons of 2005 to 2007. Several factors, which are not necessarily mutually exclusive, may account for this: first, our method of offering pupae on paper may expose solitary cocoons more to hyperparasitoids than occurs in nature. Second, when considering the total number of pupae in a brood, we offered more *C. glomerata* than *C. rubecula* pupae. Although more hyperparasitoids were recovered from gregarious pupae, the rates of individual clutches of *C. glomerata* that contained any hyperparasitoid and solitary pupae of *C. rubecula* that were hyperparasitized were similar. The hyperparasitism rates on *C. glomerata* underestimate the actual rates at which clutches were found by more than one hyperparasitoid. Third, due to the setup of this field study, we excluded hyperparasitism by primary hyperparasitoids (that oviposit in the parasitoid larvae when these develop within the caterpillars). Therefore, *L. nana* might encounter less competition from primary hyperparasitoids and may therefore alter its oviposition strategies. Fourth, as described above, female hyperparasitoids, such as *L. nana*, may exploit a large proportion of their host pupae once they locate a host clutch [31]. The hyperparasitoids locating a gregarious brood spend more time on the brood and are egg limited when exploiting the whole brood, whereas they are time limited when exploiting solitary pupae. Therefore, hyperparasitism rates may have been elevated on solitary *C. rubecula* pupae despite the larger number of eggs laid in gregarious broods.

### Plant Fitness Benefit of Volatile Release

Our study shows that enemies of those natural enemies that benefit plant fitness may also use plant-produced odours to find their hosts or prey. In this way, the plant may be caught between a “rock and a hard place,” in that two out of three trophic levels of consumers that are detrimental to the plant (either directly, through herbivory, or indirectly, through a reduction in the abundance of beneficial carnivores at the third trophic level, caused by organisms at the fourth trophic level) benefit from using herbivore-induced plant volatiles. The beneficial effect on plant fitness of attracting parasitic wasps, to indirectly defend itself against their herbivore attackers, has been intensively discussed [2],[3],[6]. Although it has been recognized that volatiles released by plants that are under attack by herbivores provide parasitoids and predators with a cue that can be used in host location, the presence of active “signalling” and associated selection on plants that are stronger signallers has thus far received less attention. Although some studies have reported a fitness benefit of plants on which herbivores were attacked by parasitoids [10]–[12], other studies have reported negative fitness consequences of plants emitting volatiles by becoming more apparent to herbivores [8]. It is important to emphasize that volatile cues may provide many community members with information and thereby may not necessarily result in a fitness benefit to plants [6]: although plant volatiles may function as a “cue” to parasitoids, they may not be a specific “signal” released by the plant (implying a selective benefit). Although short-term negative consequences of attracting hyperparasitoids for plants may be absent, as the hyperparasitoid is not affecting the direct benefit of reduced herbivory by parasitized caterpillars, the plant may be presented with a cost of reduced population size of its beneficial natural enemies when a next generation of herbivores arrives. Our results show that hyperparasitoids may parasitize up to 55% of the parasitoid offspring, therefore potentially playing a major role in parasitoid population dynamics. Furthermore, the parasitoid species studied here have been found to parasitize over 90% of the herbivores when parasitoids are at their peak abundance during the season [32]. The effect of parasitoid–hyperparasitoid interactions therefore may have significant consequences for herbivore populations, and thereby indirectly hyperparasitoids may significantly contribute to selection on plant traits such as volatile release.

### Conclusion

The fitness consequences of the emission of herbivore-induced plant volatiles are dependent on the quantitative composition of the plant-associated community at several trophic levels. The fitness benefit of volatile release should, therefore, be evaluated in the natural context of the plant-associated insect community including fourth-trophic-level organisms [6]. This will help to improve our understanding of the function of herbivore-induced volatiles in plants and of how the ecological effects of volatiles can shape the life histories of species interacting in insect communities associated with these plants [33],[34]. Furthermore, these findings are important in the context of developing Integrated Pest Management strategies in which herbivore-induced volatiles of crops are manipulated to optimize the control of insect pests by using parasitoids. Overexpression of herbivore-induced plant volatiles in crops or field application of synthetic parasitoid attractants may not benefit pest control in conditions where the responses of hyperparasitoids to HIPVs cause major mortality to parasitoids [35].

## Materials and Methods

### Plants and Insects

*Brassica oleracea* var *gemmifera* cv. Cyrus plants used for olfactometer experiments were grown in 1.45-l pots containing peat soil (Lentse potgrond, no. 4, Lent, the Netherlands) and provided with SON-T light (500 µmol/m^2^/s; L16:D8) in addition to natural daylight in a glasshouse compartment (18–26°C, 50%–70% r.h.). When plants were 4 wk old, they were fertilized weekly by applying 100 ml nutrient solution of 2.5 mg/l Kristalon Blauw (Hydro Agri Rotterdam, the Netherlands (N-P-K-Mg) 19-6-20-3) to the soil and used in experiments when they were 7 wk old.

To prepare parasitized caterpillars for the induction treatments, individual first instar *P. rapae* larvae were exposed to a single female *C. glomerata* or *C. rubecula*, which were allowed to parasitize the caterpillar in a glass vial. For *C. glomerata*, caterpillars were considered to be parasitized when the wasp had inserted her ovipositor in the caterpillar for at least 5 s. For *C. rubecula*, because of herbivore immune responses to parasitoid eggs [34], the wasp was allowed to oviposit 3 times in the same caterpillar, to increase the success rate of parasitism. Due to larval cannibalism among the parasitoids, only a single *C. rubecula* larva would develop eventually [36].

The hyperparasitoid *L. nana* was reared on *C. glomerata* cocoons in the absence of plant and herbivore-derived cues.

### Y-Tube Olfactometer Assays

All *Brassica oleracea* var *gemmifera* cv. Cyrus plants for the olfactometer assays were treated 24 h before the tests. First, plants were infested with either two unparasitized fourth instar *Pieris rapae* caterpillars or two fourth instar caterpillars that contained fully grown parasitoid larvae of either *C. glomerata* or *C. rubecula* as a result of parasitization of the caterpillar in their first instar. In a second experiment with oral secretions of caterpillars, plants were artificially damaged with a pattern wheel by drawing three lines of 3 cm long on each of the four youngest fully expanded leaves and treated with 25 µl of caterpillar oral secretions onto the damaged sites. Oral secretions were collected from healthy and *C. glomerata*–parasitized fourth instar *P. rapae* caterpillars, using 5 µl capillaries. Single caterpillars regurgitated 2–8 µl that we pooled to be used in the induction treatments. We decided not to test the relative attractiveness of plants induced with oral secretions of *C. rubecula*–parasitized caterpillars, because it would lack biological relevance as the data from our choice assays with actual feeding damage indicate that the quantity of damage by gregariously parasitized caterpillars is likely to explain the preference of hyperparasitoids for treatments with higher amounts of leaf damage (Figure 4). We have restricted to testing the effect of qualitative differences in the oral secretion of parasitized and unparasitized caterpillars only to treatment combinations where we did not identify a statistical difference in the amount of damage between the treatments.

To test whether volatiles derived from oral secretion itself may be attractive to hyperparasitoids, we applied 25 µl oral secretion of *C. glomerata–*parasitized *P. rapae* caterpillars onto undamaged plants with a fine brush. We tested the relative attractiveness of the oral-secretion-treated plants to undamaged plants treated with 25 µl of water.

Shortly before *L. nana* females were tested for their behavioural response to plant volatiles in Y-tube olfactometer bio-assays, we removed caterpillars and their feces from the plants and placed the plants in one of two glass jars (30 l each) that were connected to the two olfactometer arms. A charcoal-filtered airflow (4 l/min) was led through each arm of the Y-tube olfactometer system, and a single wasp was released at the base of the stem section (3.5 cm diameter, 22 cm length) in each test [37]. Wasps that passed a set line at the end of one of the olfactometer arms within 10 min and stayed there for at least 15 s were considered to have chosen for the odour source connected to that olfactometer arm. To compensate for unforeseen asymmetry in the setup, we swapped the jars containing the plants after testing five wasps and replaced the set of plants by a new set of plants after testing 10 wasps. The Y-tube olfactometer setup was placed in a climatized room, and in addition to daylight it was illuminated with four fluorescent tube lights (FTD 32 W/84 HF, Pope, the Netherlands).

### Field Assay Hyperparasitoid Attraction to Plant Volatiles

Eighty-four-week-old plants were transplanted into the field with 1×1 m spacing between plants and allowed to adjust to field conditions for 1 wk. Thereafter, the plants were subjected to one of four induction treatments: (1) not treated with herbivory (i.e., undamaged controls, UD), (2) infested individually with either two unparasitized first instar *P. rapae* caterpillars (PR), (3) two *C. glomerata–*parasitized *P. rapae* caterpillars (PR-CG), or (4) two *C. rubecula–*parasitized *P. rapae* caterpillars (PR-CR).

Unparasitized and parasitized caterpillars were allowed to feed on plants for 10 d, which was approximately the whole development period of the *Cotesia* larvae. Each plant was covered with a fine-mesh net when planted to avoid other herbivore infestations on the foliage and to prevent the herbivores used for induction to wander off the plant.

To test the effects of plant induction with different types of herbivory on hyperparasitism, we attached parasitoid pupae onto the plants in the field. Individual pupae of *C. rubecula*, or clutches of *C. glomerata*, were first attached to a paper disc (3×3 cm) with a small droplet of glue. We removed nets and caterpillars just before attaching the paper discs carrying the pupae with a pin needle. Half of the plants for each treatment received five *C. glomerata* clutches, and the other half received five *C. rubecula* pupae. The pupae were exposed to the natural community of hyperparasitoids and recollected after 5 d. They were kept separately in 2.2 ml Eppendorf tubes that were closed with cotton wool. The Eppendorf tubes were checked daily for emerging primary parasitoids and hyperparasitoids. All wasps were identified to species level.

A completely randomized design was applied to the field studies. Five replications, each with 80 plants (10 replicates of each treatment), were carried out from June until October 2011.

### Field Collections of Parasitoid Cocoons

To assess hyperparasitism rates and species communities on the solitary and gregarious primary parasitoid, we established plots of 6×6 m containing 49 plants of *B. oleracea* cultivars in an experimental field in the vicinity of Wageningen, the Netherlands, during 3 consecutive years (2005–2007). Within plots, plants were planted in a square of 7×7 plants with a spacing of 75 cm between plants. Plots were isolated by strips of 6 m wide that were sown with a grass mixture of *Lolium* and *Poa* species. During the growth season of cabbage plants, from early May until the end of September, we conducted weekly surveys on the plants for *C. glomerata* and *C. rubecula* pupae by investigating both sides of all leaves of all plants in the plots. The pupae were collected and placed individually in 2.2 ml Eppendorf tubes and closed with cotton wool. The pupae with their external silk cocoon were weighed, and for the gregarious *C. glomerata*, the brood size was determined. The Eppendorf tubes were checked daily for emerging parasitoids, which were individually transferred to another Eppendorf tube and stored at −20°C. All wasps were identified to species level.

### Plant Volatile Analysis

#### Headspace collection of plant volatiles

To characterize the differences in plant volatile release after herbivory by unparasitized and parasitized caterpillars, we collected the headspace of 6-wk-old *B. oleracea* plants subjected to different herbivore treatments. Dynamic headspace sampling was carried out in a climate room, and we collected 12 replicates of each of four experimental treatments: (1) undamaged plants, plants infested with two *P. rapae* caterpillars that were either (2) unparasitized or parasitized by (3) *C. glomerata* or (4) *C. rubecula*. Caterpillars were allowed to feed for 7 d, during which the parasitoid larvae nearly completed their development inside the herbivore. Prior to volatile collection, caterpillars were removed and plants were removed from their pots. Their roots and soil were carefully wrapped with aluminium foil. During volatile collection, the plants were placed individually into a 25 litre glass jar, which was sealed with a viton-lined glass lid with an inlet and outlet. Compressed air was filtered by passing through charcoal before reaching to the glass jar containing the plant. Volatiles were trapped by sucking air out of the glass jar at a rate of 150 ml min^−1^ through a stainless steel tube filled with 200 mg Tenax TA (Markes, Llantrisant, UK) for 4 h.

#### Analysis of plant volatiles

A Thermo Trace GC Ultra coupled with Thermo Trace DSQ quadrupole mass spectrometer (Thermo Fisher Scientific, Waltham, MA, USA) was used for separation and detection of plant volatiles. Prior to releasing the volatiles into the GC, the Tenax TA cartridges were dry-purged under a stream of nitrogen (20 ml min^−1^) for 10 min at ambient temperature in order to remove moisture and oxygen. The collected volatiles were released from the Tenax TA thermally on an Ultra 50∶50 thermal desorption unit (Markes, Llantrisant, UK) at 250°C for 10 min under a helium flow of 20 ml min^−1^, while re-collecting the volatiles in a thermally cooled universal solvent trap at 10°C using Unity (Markes, Llantrisant, UK). Once the desorption process was completed, volatile compounds were released from the cold trap by ballistic heating at a fast rate (40°C s^−1^) to 280°C and was then kept at 280°C for 10 min, while the volatiles transferred to a ZB-5MSi analytical column (30 m×0.25 mm I.D. ×1.00 µm F.T.; Phenomenex, Torrance, CA, USA) in a splitless mode for further separation. The column was operated at an initial temperature of 40°C and the temperature was raised at 5°C min^−1^ to 280°C and held for 4 min under a column flow of 1 ml min^−1^ in a constant flow mode. The DSQ mass spectrometer (MS) was operated in a scan mode with a mass range of 35–350 amu at 5.38 scans s^−1^, and ionization was performed in EI mode at 70 eV. The MS transfer line and ion source were set at 275 and 250°C, respectively. Compound identification was based on comparison of mass spectra with those in the NIST 2005 and Wageningen Mass Spectral Database of Natural Products MS libraries. Experimentally obtained linear retention indices (LRI) were also used as additional measures for confirming the identity of compounds. Relative quantification by peak areas of individual compounds was done using the integrated absolute signal of a quantifier ion in the selected ion monitoring (SIM) mode. The individual peak areas of each compound were computed into peak area per gram shoot biomass to correct for differences in size of individual plants.

### Statistical Analysis

*L. nana* preferences for herbivore-induced plant volatiles, as tested in two-choice Y-tube olfactometer assays, were analysed using two-tailed binomial tests.

Hyperparasitoid preferences for plant volatiles induced by unparasitized *P. rapae* caterpillars and caterpillars parasitized by gregarious or solitary primary parasitoids under field conditions were analysed using two Generalized Linear Models (GLMs). To analyse the effects of plant inductions with different types of herbivory on hyperparasitism at plant level, we modelled the dependent variable as a binomial occurrence of hyperparasitism per plant (400 plants equally divided over five replicates) and scored presence of hyperparasitoids in pupae as 1 and absence as 0. Additionally, to test the effects of the plant inductions on hyperparasitism at cocoon level, we modelled the dependent variable as the number of pupae or clutches giving any hyperparasitoid out of the fixed totals of five pupae attached to the plant. Into the two models we included the fixed factors caterpillar induction (undamaged, unparasitized *P. rapae*, *P. rapae* parasitized by *C. glomerata*, and *P. rapae* parasitized by *C. rubecula*), replicate (five replications), types of pupae (gregarious or solitary), and the interactions between the three terms.

For the field collections of solitary and gregarious pupae (*n* = 1,668 and 1,256, respectively), we analysed whether the gregarious broods of primary parasitoids were more frequently found by hyperparasitoids than solitary pupae and whether this results in differences in the total fraction of hyperparasitism of primary parasitoid offspring from gregarious and solitary species. First, we tested whether solitary and gregarious pupae of the primary parasitoids differed in the proportion of occasions that these were found by a hyperparasitoid. For each solitary parasitoid, we scored a 1 when there was a hyperparasitoid emerging and a 0 when the primary parasitoid emerged. Because gregarious broods could be hyperparasitized to different degrees (percentage pupae parasitized), which may be a result of a single hyperparasitoid finding the gregarious brood and parasitizing several pupae or a result from multiple occasions on which hyperparasitoids found the gregarious brood, we scored a 1 when any hyperparasitoid emerged from the gregarious *Cotesia* brood. We used a Generalized Linear Model (GLM) to test for the effect of *Cotesia* species and year as well as their interaction on the binomially distributed occurrence of hyperparasitism.

Second, we tested whether hyperparasitoids exert different levels of parasitism of gregarious and solitary primary parasitoids. Within each of the years, for each *Cotesia* species we counted the total number of emerging wasps for each of the primary parasitoid and hyperparasitoid species. The data on species composition and their abundance per *Cotesia* species were subjected to Chi-square tests to assess parasitoid community differences for primary parasitoid brood size (solitary or gregarious). One of the hyperparasitoid species (i.e., *Baryscapus galactopus*) parasitizes the larvae of *Cotesia* when these are still inside a caterpillar and lay several eggs within a single *Cotesia* larva. The *B. galactopus* brood develops when the *Cotesia* larvae spin their cocoon outside the caterpillar and *B. galactopus* wasps emerge from the *Cotesia* pupa. The brood size of *B. galactopus* on *Cotesia* is on average eight *B. galactopus* per *Cotesia* pupa, and therefore, we recalculated the total incidence of parasitization of *Cotesia* pupae by dividing the *B. galactopus* numbers by 8 and rounding off to the nearest whole number (numbers of each parasitoid species are presented in Table 5). All statistical tests were performed with the statistical software package Gen Stat (10^th^ edition).

We used Partial Least Squares Projection to Latent Structures-Discriminant Analysis (PLS-DA) to analyse which of the compounds contributed most to describing the difference among plant treatments. The compounds that scored >1 in their Variable Importance in the Projection (VIP) scores were subjected to Mann–Whitney U tests among treatment pairs to test for significant differences among treatments.

## Abbreviations

CG: *Cotesia glomerata*
CR: *Cotesia rubecula*
(*E*)-DMNT: (*E*)-4,8-dimethylnona-1,3,7-triene
PR: *Pieris rapae*
UD: undamaged
